# Is lactate the new panacea for endothelial dysfunction?

**DOI:** 10.1186/s13054-014-0614-x

**Published:** 2014-12-01

**Authors:** Marek Nalos, Benjamin M Tang, Ralph Nanan

**Affiliations:** Department of Intensive Care Medicine, Sydney Medical School - Nepean, University of Sydney, Nepean Hospital, Derby Street, Penrith, NSW 2750 Australia; Department of Pediatrics, Sydney Medical School - Nepean, University of Sydney, Nepean Hospital, Derby Street, Penrith, NSW 2750 Australia

## Abstract

Fluid resuscitation in the critically ill is a hot topic. The current strategy of rapid and adequate resuscitation in shock followed by conservative fluid administration is often difficult to achieve with standard crystalloid solutions. Research into alternative intravenous fluids tailored to individual patient needs is required. In the previous issue of *Critical Care*, Somasetia and colleagues compare the effects of hypertonic sodium lactate with the World Health Organization-recommended strategy of Ringer’s lactate resuscitation in children with severe Dengue, a viral infection for which causal treatment and vaccination are not available. The results not only suggest unimpaired lactate metabolism during shock in children but document improvement in endothelial barrier function, limited coagulopathy, and avoidance of fluid overload with hypertonic sodium lactate. Their study invites several important questions to be answered. Is hypertonicity or lactate *per se* important for the beneficial effects? Are the metabolic or anti-inflammatory effects responsible? Is the raised lactate in shock an adaptive response? Should reduction in lactate levels be the goal of resuscitation? These questions may trigger further research into the role of lactate and lactate-based intravenous fluids in resuscitation of the critically ill.

Dengue fever is the most prevalent mosquito-borne viral illness in humans. In tropical and subtropical countries, an estimated 500,000 patients, mostly children, require hospitalization each year, with overall mortality rate around 2.5%, however reaching up to around 40% in cases with established shock [1,2]. There are four serotypes of the enveloped, single-stranded RNA Dengue virus. Immunity following infection with one serotype does not protect the host from being infected by another serotype and in fact may trigger more severe disease [3,4].

The hallmarks of severe Dengue infection, also termed Dengue hemorrhagic fever (DHF) and Dengue shock syndrome (DSS), are mucosal bleeding and vascular leakage syndrome due to complex interplay between the virus, platelets, and immune and endothelial cell activation leading to barrier dysfunction, plasma leakage, hemoconcentration, thrombocytopenia, and coagulopathy [5]. The profound vascular leakage leads to circulatory collapse and accumulation of interstitial fluid with respiratory, cardiac, hepatic, and cerebral function impairment [2]. The molecular events triggered by the virus lead to endothelial activation with type I interferon and pro-inflammatory cytokine production associated with swelling of endothelial cells and shedding of adhesion molecules such as vascular cell adhesion molecule-1 (VCAM-1) [6]. Although it has been proposed that soluble VCAM-1 (sVCAM-1) levels may reflect severity of DHF/DSS, this remains unproven [7].

The World Health Organization (WHO) recommends that patients with DHF/DSS be treated with an immediate volume replacement using isotonic crystalloid solutions, followed by the use of plasma or colloid solutions for profound or continuing shock [2]. The goals of fluid resuscitation include decreasing tachycardia and improving pulse volume, capillary refill time, and end-organ perfusion while correcting metabolic acidosis. During the critical and recovery phase, however, excessive fluid therapy is associated with pulmonary edema, congestive heart failure, massive pleural effusion, and ascites. In this respect, the study by Somasetia and colleagues in the previous issue of *Critical Care* is a pioneering work [1]. They randomly assigned 50 children either to the standard WHO recommendation-based protocol of fluid resuscitation with Ringer’s lactate (28 mmol/L) or to hypertonic sodium lactate (HSL) solution that contains 504 mmol/L of lactate. Apart from reaching their primary endpoint of reduction in sVCAM-1, suggesting reduced inflammatory activation of the endothelium, the lactate-based regime was associated with several important effects.

First, the volume of fluid required in the HSL group was much lower during the 12-hour intervention period to the extent that the overall fluid balance was neutral. This may be related to the following: (a) HSL is a hypertonic solution that may draw interstitial fluid back into the vasculature; (b) lactate as a metabolizable anion may lead to chloride egress from endothelial cells, causing reduction in swelling and improved barrier function [8]; and (c) it was recently shown that lactate by itself has important anti-inflammatory properties that may have reduced inflammatory response and endothelial activation [9].

Second, plasma lactate levels actually fell in the HSL group despite a substantial amount of infused lactate. This suggests that these critically ill children had unimpaired lactate metabolism. Consequently, the high strong ion difference of the HSL solution resulted in metabolic alkalosis and hypokalemia, which were similar to HSL effects reported in a study by our group [10].

Third, although more rescue starch boluses were given in the observation period (second 12 hours), suggesting that the effect of HSL is short-lived, less blood products were required, implicating a reduction in coagulopathy. The reason for this is unclear but may be related to less hemodilution and higher pH associated with HSL infusion.

The study by Somasetia and colleagues was a pilot study with sVCAM-1 as a primary surrogate outcome of limited clinical importance [7]. Despite this, the authors made a substantial contribution to the important field of fluid resuscitation in sepsis, particularly in the pediatric population. The Fluid Expansion as Supportive Therapy (FEAST) study has already challenged the concept that large-volume fluid resuscitation is beneficial in sepsis [11], and this study suggests that by using HSL solution instead of standard fluid therapy, a smaller volume is required, and this could be associated with less coagulopathy while providing useful energetic substrate for vital organs, reduce inflammation, correct acidosis, and end up with a reasonable fluid balance avoiding the ‘Michelin man’ effect (increased interstitial fluid accumulation and peripheral edema). Although the FEAST trial concerned fluid resuscitation in bacterial sepsis and malaria, which have pathogeneses different from those of DHF, endothelial cell dysfunction is common to all. So one may agree with the authors: ‘from a theoretical point of view, the optimal treatment should address both the cause (endothelial dysfunction) and consequence (hypovolemia)’ of increased vascular permeability [1]. This work thus may prompt trials of hypertonic lactate resuscitation in sepsis with the aim to restore vascular permeability. Nevertheless, one needs to take into account the potentially deleterious effects of lactate on immune function in sepsis [12].

## Abbreviations

DHF: Dengue hemorrhagic fever
DSS: Dengue shock syndrome
FEAST: Fluid Expansion as Supportive Therapy
HSL: hypertonic sodium lactate
sVCAM-1: soluble vascular cell adhesion molecule-1
VCAM-1: vascular cell adhesion molecule-1
WHO: World Health Organization
